# Short-Term Hermetic Storage of Wet Maize and Its Effect on Quality

**DOI:** 10.3390/foods12040891

**Published:** 2023-02-19

**Authors:** Nileshwari Raju Yewle, Richard L. Stroshine, RP Kingsly Ambrose, Dieudonne Baributsa

**Affiliations:** 1 Department of Agricultural Engineering, Visva-Bharati University, Sriniketan 731236, West Bengal, India; 2 Department of Entomology, Purdue University, West Lafayette, IN 47907, USA; 3 Department of Agricultural and Biological Engineering, Purdue University, West Lafayette, IN 47907, USA

**Keywords:** postharvest management, humid tropics, drying, grain storage, germination

## Abstract

Maize is a major crop grown in many regions of the world for human consumption, starch production, and animal feed. After harvest, maize is dried to avoid spoilage caused by fungal growth. However, in the humid tropics, drying maize harvested during the rainy season poses challenges. In such instances, temporary storing maize under hermetic conditions may preserve grain quality while waiting for conditions suitable for drying. Wet maize at the moisture contents (m.c.) of 18, 21, and 24% was stored for up to 21 days in both hermetic and non-hermetic jars. The stored maize was assessed, every 7 days, for germination and related parameters, presence of visible mold, and pH. After 21 days of storage at 18, 21, and 24% m.c., maize germination decreased by 28.5, 25.2, and 95.5 percentage points, respectively, in hermetic jars; and by 28.5, 25.2, and 94.5 percentage points in non-hermetic jars (control). There was visible mold on maize stored in non-hermetic jars after 21 days regardless of m.c. Maize at 21 and 24% m.c. stored in hermetic conditions underwent lactic acid fermentation that reduced the pH. The findings suggest that maize at 18 and 21% m.c. can be stored for 14 and 7 days, respectively, under hermetic conditions without significant loss of quality. Further research is needed to thoroughly assess the application of these findings for temporarily storing and subsequently drying maize on farms and along the grain value chain.

## 1. Introduction

Maize, an important staple crop cultivated by millions of smallholder farmers, is used for animal feed and can be processed into food and industrial products [1,2]. Worldwide maize production, the highest among cereals, was estimated at 1129 million metric tons during the 2020–2021 crop season [3]. Despite its importance, a significant portion of this maize is lost during postharvest in developing countries due to biotic and abiotic factors including pests and mold [4,5,6]. Postharvest losses in Africa range from 14–36% and occur along the maize value chain including harvesting, drying, threshing and shelling, transport, storage, and commercialization [7]. Insects play a major role in contributing to maize losses as they damage grain pre- and postharvest [8].

Several approaches have been used to minimize grain postharvest handling and storage losses, including drying and storage technologies. However, smallholder farmers in developing countries rely mostly on traditional methods to mitigate postharvest losses. For example, sun drying is done in the field and continues at the homestead to reduce grain moisture content (m.c.), prevent fungal growth and severe insect infestations, and maximize storage life [9,10,11]. Traditional and improved storage methods (e.g., treatment with ash, pesticides or botanicals, and hermetic bags) are used to maintain grain dryness and or control storage insect pests [12,13,14].

Drying grain after harvest in the humid tropics is a major challenge for smallholder farmers due to unfavorable weather conditions such as rain or cloud cover. Most smallholder farmers rely on the sun to dry their crops due to limited access to appropriate drying equipment [10]. Sun drying is done by spreading the cobs or grain on the ground, mats, tarpaulins, or plastic sheets [10,11]. Hence, drying crops in the humid tropics is a slow and labor-intensive process. Several consecutive days of rain and cloud cover can make it difficult to sun dry a crop and result in the loss of grain quality. Qualitative loss includes changes in appearance, nutritional degradation, loss of germination capacity, and the presence of insect and mold contamination [15].

Studies have assessed hermetic storage of wet maize to preserve grain quality [15,16,17]. Though the use of hermetic storage technologies (e.g., Purdue Improved Crop Storage- PICS, GrainPro SuperGrainbag) may prevent losses due to fungal and insect pests on dried grain [16,18,19,20,21], they have resulted in a loss of quality when wet maize was stored for a month or more. Hermetic storage of wet grain can result in high respiration rates of grain which may compromise germination as well as other quality factors (e.g., discoloration) [6,22,23]. Maintaining grain quality, even when maize is wet, is vital not only to supplying food and feed that is free of contaminants but also seed. In many developing countries smallholder farmers use a portion of the stored crop as seed for the next crop.

Though maize germination may be high at the time of harvest, maintaining seed viability requires proper storage conditions. Short-term hermetic storage of wet maize could be a viable option for smallholder farmers in the humid tropics when rain and cloud cover delay the drying process. A study conducted to assess the storage of wet maize in hermetic bags showed a reduction by a third and a complete loss of germination of maize at 18 and 21% m.c., respectively, after 30 days of storage [16]. Similarly, in-vitro hermetic storage of maize at 22% m.c. showed a decrease in germination by about a third after 15 days [15].

Evaluating temporary (several days to several weeks) hermetic storage of wet maize, during drying, is needed to determine the optimal storage time with minimal effect on grain quality. This study was initiated to investigate how germination and other qualities change during the first three weeks of hermetic storage of wet maize. Unlike previous studies that explored the use of hermetic storage to avoid drying grain for use in animal feed and for industrial purposes [15,24], this study assessed the efficacy of temporarily holding wet maize in hermetic containers after harvest (for human consumption or planting), when drying conditions are not optimal. The findings of this research can significantly help smallholder farmers and other grain value chain actors in developing countries to minimize postharvest losses and improve food and nutritional security.

## 2. Material and Methods

### 2.1. Grain Sample Preparation

Ears of maize (*Zea mays*, variety Becks 5994V2P) were harvested by hand from a plot at the Agronomy Center for Research and Education (ACRE, Purdue University West Lafayette, IN, USA) on 5 October 2021. The kernel moisture content (m.c.) was approximately 20% when the maize was shelled (the next day) using an Agriculex SCS-2 single-ear corn sheller (Guelph, Ontario, Canada). After shelling, the maize was cleaned in a benchtop mini-Clipper grain cleaner (Blount Ferrell Ross, Bluffton, Indiana), and then kept in heavy-duty plastic bags stored at 4 °C. Several days before the experiment began, the m.c. was determined to be 19.6% by the oven-dry method (Standard S352, ASABE, 2017). The grain was divided into three batches. Two batches were re-wetted to targets of 21 and 24% m.c. using a Morse variable speed drum roller Model 1-5154 VS (East Syracuse, NY, USA) while the third was air-dried to 18% m.c. After drying and rewetting, maize m.c.± standard deviations were 17.59 ± 0.02% for 18%, 21.03 ± 0.03% for 21%, and 24.08 ± 0.07% for 24%.

### 2.2. Experimental Setup and Gas Monitoring

The experiment was conducted in the PICS laboratory at Purdue’s Department of Entomology (19 November to 10 December 2021). About 700 g of maize was placed in a 1-L round reusable Pyrex glass jar (Corning Inc., Tewksbury, MA, US). There were two storage conditions- hermetic and non-hermetic. The non-hermetic storage was considered a control. For the hermetic storage, the jars were sealed with a metal lid wrapped with Parafilm™ to ensure an airtight seal; while for the non-hermetic storage, the covering of the jars consisted of a disc of Whatman filter paper (Grade 1, 47 mm Circle) held in place by the ring normally used with metal covers. The filter paper allowed gas exchange with the surrounding environment. There were four replications for each combination of the three m.c. (18, 21, or 24%), two storage methods (hermetic or non-hermetic), and three storage times (7, 14, or 21 days), giving a total of 72 jars. The jars were placed in a Caron Insect Growth Chamber (Model 6025–1, 115 VAC, Caron Growth chambers, OH, USA) maintained at 25 °C and 80% relative humidity (r.h.).

To enable gas measurement in the hermetic jars, a hole was drilled in the lid and sealed with a silicon-rubber septum. Both oxygen (O_2_) and carbon dioxide (CO_2_) levels were measured using a MOCON^®^ portable oxygen/carbon dioxide analyzer (Pac Check^®^ 325, Mocon Inc., Brooklyn Park, MN, USA). Gas measurements were taken every 12 h up to 21 days for both hermetic and non-hermetic jars.

### 2.3. Data Collection on Maize Samples

Initial assessments were conducted using four maize samples taken from each batch at the targeted m.c. before the grain was placed in the jars. For each m.c. and storage method combination, four jars were opened 7, 14, and 21 days after the start date of the experiment to collect data.

#### 2.3.1. Germination and Seedling Growth Assessment

Maize germination was determined using the International Rules for Seed Testing protocol [25]. Four 25 kernel samples were taken from each jar giving a total of 100 kernels. Each day, for a week, kernels were assessed for germination. A kernel was considered to have germinated when the shoot was at least 2 mm. To assess seedling growth, ten germinated seedlings were randomly selected from each sample at the end of the germination period. The shoot (SL) and root lengths (RL) were measured with a caliper [26]. The values were summed and divided by the number of seeds that germinated to determine the average root and shoot length.

#### 2.3.2. Moisture Content (m.c.)

Maize m.c. was determined using the oven dry method at 103 ± 1 °C [27] by assessing weight before and after drying. Three 20 g samples of grain were dried for 72 h.

#### 2.3.3. pH Measurement

The pH of each sample was determined using a pH meter (Denver Instrument Company, Goettingen, Germany). Seventy grams of distilled water were added to seventy grams of maize that had been placed in a 500 mL beaker. A magnetic mixer was used to stir the contents of the beaker for 50 s, and then the liquid was poured into a clean 15 mL centrifuge tube. The pH reading was recorded after 30 s. All measurements were taken in triplicate.

#### 2.3.4. Visual Mold Assessment

At each sampling, roughly 250 g of kernels were taken from each jar using a four-slot probe. A sample of 25 kernels from each jar was examined for signs of fungal growth with the naked eye and also using a stereomicroscope [16,22]. A sub-sample of 6–8 maize kernels from each sample of 25 kernels was assessed for visible mold using a Leica S6 D Greenough stereo microscope (Leica Microsystems, Wetzlar, Germany) at a 10× magnification.

### 2.4. Data Analysis

The statistical analyses were performed using SAS Software version 9.4. A two-way ANOVA was used to compare data of the three m.c. (18%, 21%, and 24%) measured at the four storage times (0, 7, 14, and 21 days). The Student–Newman–Keuls (SNK) method was used to compare means. In addition, using the data for all the storage times and m.c., the Pearson correlation coefficients were computed for all variables.

## 3. Results

### 3.1. Germination, Shoot Length, and Root Length

**Figure 1:**
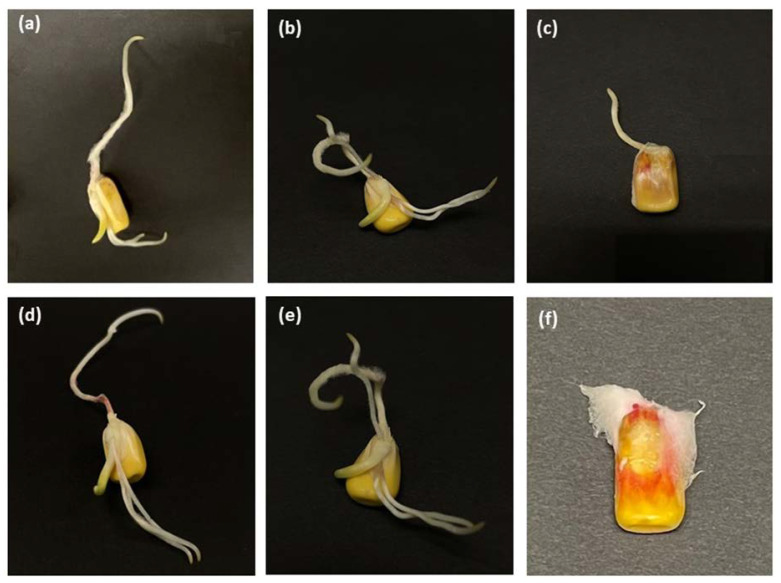
Pictures of maize kernels were magnified using a stereomicroscope (10×). Germinated kernel samples are for maize at 18, 21, and 24% moisture content stored for 21 days in non-hermetic (**a**–**c**) and hermetic (**d**–**f**) jars, respectively.

The results for germination, shoot length, and root length are summarized in Table 1. Differences in germination rates were significant among hermetic and non-hermetic storage methods (F = 34.94; df = 15; *p* = 0.001). Germination rates of maize at 18 and 21% m.c. stored in both hermetic and non-hermetic jars were greater than 90% after 14 days of storage. However, it decreased to between 68 and 88% after 21 days. At 24% m.c., germination rates of maize stored in both hermetic and non-hermetic jars decreased as storage time increased, dropping to below 5% after 21 days. There was considerable variability in both shoot and root length (Table 1 and Figure 1). The average shoot lengths were similar among treatments within 7 and 14 days. At 21 days, shoot lengths of the maize at 18 and 21% m.c. were significantly greater than for maize at 24% m.c. stored in both hermetic and non-hermetic jars. The average root lengths were similar among treatments within 7 days but varied at 14 and 21 days with no clear pattern.

Two-way ANOVA indicated statistical differences for both storage methods and time. Maize germination rates were affected by both storage time (F = 327.39; df = 5; *p* = 0.001) and m.c. levels (F = 66.64; df = 3; *p* = 0.001). Changes in shoot length were statistically significant and affected by both m.c. (F = 3.87; df = 3; *p* = 0.001) and storage time (F = 19.09; df = 5; *p* = 0.001). The differences in root length were also statistically significant for the m.c. treatments (F = 17.10; df = 3; *p* = 0.001) and storage time (F = 59.87; df = 5; *p* = 0.001). The Pearson correlations revealed several relationships between germination and both root and shoot length. There was a positive correlation between shoot length and percent germination with Pearson correlations of 0.652 and 0.440 (*p* > 0.01) for hermetic and non-hermetic storage, respectively. There was also a strong positive correlation for root length and percent germination with Pearson correlations of 0.746 and 0.753 (*p* > 0.01) for both hermetic and non-hermetic storage, respectively.

### 3.2. Gas Composition in the Jars 

**Figure 2:**
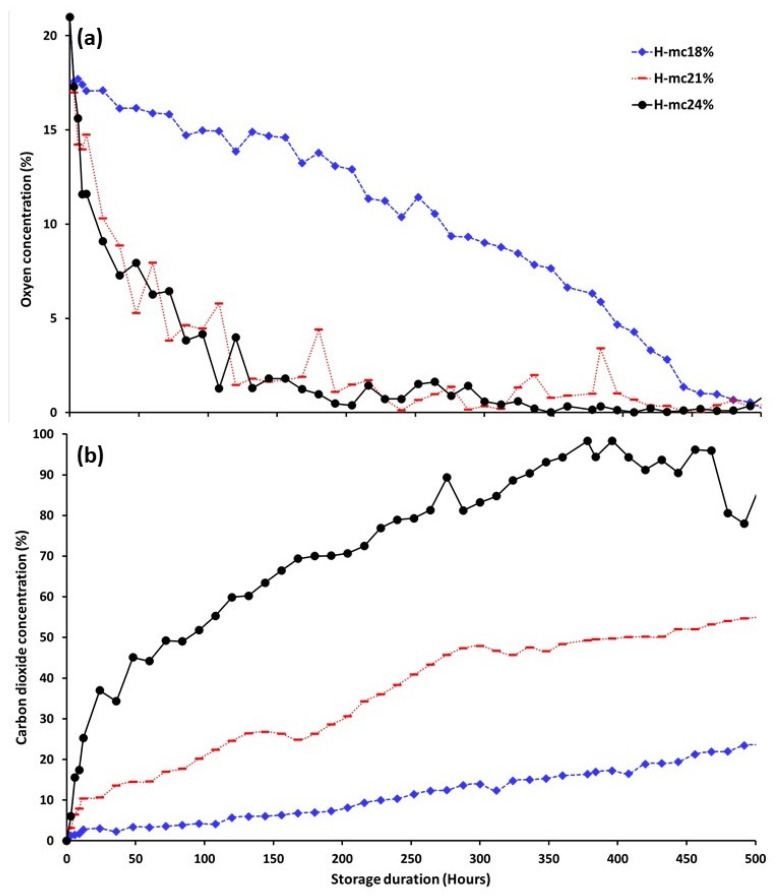
Oxygen (**a**) and carbon dioxide (**b**) levels in hermetic jars containing maize stored for up to 21 days at 18, 21, and 24% moisture content.

Figure 2 shows the levels of O_2_ (a) and CO_2_ (b) in the hermetic jars containing maize at each m.c. Oxygen levels dropped rapidly for maize at the two higher moisture contents. To reach a 5% O_2_ level, it took about 3 days for maize at 21 and 24% m.c., and 17 days for maize at 18% m.c. To reach below the 1% O_2_ level, maize at 18% m.c. stored in hermetic jars took more than twice the time of maize at 21 and 24% m.c. Although O_2_ levels in the hermetic jars at the two highest m.c. were similar, there was slightly more O_2_ in the jars containing maize at 21% m.c. compared to those at 24% m.c. The two-way ANOVA indicated that O_2_ depletions in hermetic jars were affected by both maize m.c. (F = 2365.6; df = 2; *p* = 0.001), and storage time (F = 959.84; df = 3; *p* = 0.001). There were no changes in the O_2_ levels in the non-hermetic jars (graph not shown). After 21 days, oxygen levels in non-hermetic jars were 18.5, 18.4, and 18.6% in the maize at 18, 21, and 24% m.c., respectively.

Carbon dioxide levels increased over time in all the hermetic jars. The increase was proportional to the m.c. with the highest CO_2_ level observed in the jars with maize at 24% m.c. The increases were nearly linear in the jars containing maize at 18 and 21% m.c. For the maize at 24% m.c., the CO_2_ level increased quickly during the first 60 h., and then began to increase linearly until 420 h. The two-way ANOVA indicated that CO_2_ levels in hermetic jars were affected by both m.c. (F = 2214.5; df = 2; *p* = 0.001), and storage time (F = 711.8; df = 3; *p* = 0.001). In non-hermetic jars, CO_2_ levels increased from 0.30 to 0.43% for maize at 18% m.c., 0.62 to 0.67% for maize at 21% m.c, and from 0.30 to 0.70% for maize at 24% m.c. after 21 days of storage.

### 3.3. Grain Moisture Content

No significant differences were observed within each grain m.c. between hermetic and non-hermetic treatments (F = 1.18; df = 3; *p* = 0.308) during storage (Table 2). Significant differences were observed among m.c. within each storage method (hermetic or non-hermetic) (F = 1862.14; df = 3; *p* = 0.001). As indicated earlier, maize germination rates were affected by both m.c. and storage time. There was a significant negative correlation between grain m.c. and percent germination with a Pearson correlation of −0.327 (*p* = 0.003) and −0.414 (*p* = 0.003) for hermetic and non-hermetic storage, respectively.

### 3.4. pH

The pH measurements were acidic, even before the maize was placed in storage. After 21 days, the pH of maize stored in hermetic jars decreased, while no clear trend was observed in non-hermetic jars. For maize at 21 and 24% m.c. stored in hermetic jars, the pH consistently decreased until the end of storage (Table 2). ANOVA indicated that pH was affected by both m.c. (F = 181.20; df = 3; *p* = 0.001) and storage time (F = 198.24; df = 5; *p* = 0.001). There was a significant positive correlation between pH measurements and percent germination, with Pearson correlation coefficients of 0.79 (*p* = 0.01) and 0.28 (*p* = 0.05) for hermetic and non-hermetic storage, respectively.

### 3.5. Mold Assessment

**Figure 3:**
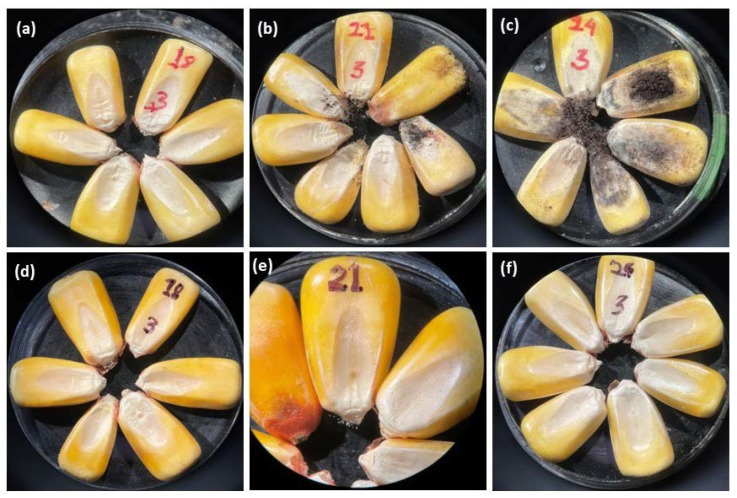
Pictures of maize kernels were magnified using a stereomicroscope (10×). Samples are for visual mold assessment of maize at 18, 21, and 24% moisture content stored for 21 days in non-hermetic (**a**–**c**) and hermetic (**d**–**f**) jars, respectively.

Mold growth decreases the quality of stored grains and seeds. At each of the storage times, we visually examined maize kernels to determine whether there was fungal growth. The greatest amounts of mold growth were observed after 21 days on maize at 21 and 24% m.c. stored in non-hermetic jars (Figure 3b,c). In addition, maize at 21 and 24% m.c. stored in non-hermetic jars produced an odor associated with spoilage. When maize was poured from these jars, there was a visible dust-like cloud emanating from the mouth of the jars. For hermetic storage at 18% m.c., there was no evidence of mold growth on maize after 21 days. However, there were several white fungal mycelia on the tip caps of the kernels of maize at 21 and 24% m.c. that first became visible after 14 days of storage. A fermented odor became noticeable in maize at 24% m.c. stored in hermetic jars after only 7 days. After 14 days, kernel discoloration (lightening or whitening of the yellow color) which increased with time in storage was observed on maize at 21 and 24% m.c. kept in hermetic jars.

## 4. Discussion

The purpose of this study was to investigate the effect of short-term hermetic storage of wet grain on the quality of maize. Smallholder farmers in the tropics need to find alternative ways of preserving maize quality while waiting for optimum conditions to dry their crops. Most smallholder farmers rely on stored grain as a source of food and seed [9]. Hence, it is crucial to assess whether and when a potential short-term hermetic storage solution for wet maize would compromise germination and other grain quality attributes such as mold growth. The targeted grain moisture contents (i.e., 18, 21, and 24%) remained constant throughout the storage times for each moisture and storage method combination. Therefore, any changes seen in maize stored at various moisture contents were due to treatment effects. This is consistent with other studies that have indicated no or minimal change in the m.c. of grain held under hermetic conditions while it changed under non-hermetic conditions [17,28,29].

### 4.1. Effect of Short-Term Hermetic Storage of Wet Maize on Germination and Physiological Growth

The initial germination of the maize used in this study was 95% or greater. Percent germination decreased with time for all moisture contents, regardless of the storage method. Within each storage time (7, 14, and 21 days), there was a statistically significant decrease in germination only in maize at 24% m.c. stored in both hermetic and non-hermetic jars. Compared to the initial values, the reductions in the germination of maize at 24% m.c. stored in both hermetic and non-hermetic were below 15 percentage points within the first 14 days. However, compared to maize at 18 and 21% m.c. after 21 days, the reductions in the germination of maize at 24% m.c. reached 72 and 84 percentage points in hermetic and non-hermetic storage, respectively. Within each moisture content after 21 days, when compared to the initial, maize germination at 24% m.c. dropped by 95.5 percentage points (no seed germinated) and 91.5 (only 4% germinated) percentage points in hermetic and non-hermetic storage, respectively. Maize at 24% m.c. stored under hermetic conditions started losing its viability during the first 7 days. 

Maize seeds are metabolically active and respire, absorb oxygen, and release carbon dioxide [23]. Previous studies reported a rapid decrease in germination rates of maize stored under hermetic conditions as m.c. increased [15,16]. Weinberg et al. (2008) [15] reported that after 15 days of storage, there was a drastic germination difference of 58 percentage points between maize stored at 18 versus 22% m.c. In our experiment for maize stored hermetically for 14 days, there was about a 3-percentage point decrease in germination when moisture increase from 18% to 21% m.c., a difference that was not statistically significant. A possible explanation is that the quality of the maize used in the two experiments was different (low initial germination rates of 28.6% at 22% m.c. and 76.0% at 18% m.c. in Weinberg’s experiment, while it was above 95% in our experiment) and the temperature at which the experiment was conducted (30 °C, which was 5 °C higher than our experiment).

There was a similar trend of a decrease in germination rates between hermetic and non-hermetic storage methods, except for maize at 24% stored for 7 days under hermetic conditions (which had a greater decrease in germination). The decrease in the germination of maize stored in hermetic jars may be due to low oxygen availability at higher m.c. [16,17]. The depletion of O_2_ to less than 5% within the first three days and the increase in CO_2_ in hermetic jars at 24% m.c. may have affected the germination rates. On the other hand, the drastic decrease in germination (from 95.5 to 4%) of maize stored in non-hermetic conditions at 24% after 21 days was probably caused by fungal growth [30]. 

Mold growth was relatively slow at 18% and 21% m.c. (Figure 3a,b), and therefore fewer kernels were affected when maize was stored in the non-hermetic jars. Although fungal growth on high-moisture maize may not be visible to the naked eye, it can damage the embryos and eventually reduce germination [31]. Unlike non-hermetic methods, hermetic storage inhibits fungal growth [16]. Based on these findings, a germination rate above 90% can be achieved for maize stored in hermetic containers for 14 days at 18 and 21% m.c. Unlike studies where grain was stored for longer periods [15,16,17], these results suggest that farmers can hermetically store maize at up to 21% m.c. or below for a short term (up to 14 days) and still maintain good germination.

Root and shoot lengths were measured to assess the vigor of the germinated seeds. Good quality seeds that germinate will have faster-growing roots and shoots [32]. A significant decrease in the average root or shoot length may indicate a detrimental effect of storage at higher moisture levels on the vigor of the seedling. The effect of storage on the shoot and root lengths was most obvious after 21 days where the shoots and roots of maize at 24% m.c. kept in hermetic and non-hermetic jars had no or minimal growth (average lengths ranging from 0 to 2.81 mm). The same trend was observed for germination rates of maize at 24% m.c. stored in hermetic and non-hermetic jars for 21 days. Low levels of O_2_ and high levels of CO_2_ in maize at 24% stored in hermetic jars and the invasion of the maize kernels by fungi that occured in non-hermetic jars may have damaged the germs, resulting in slow root and shoot growth during germination. Given the variability in the root and shoot length measurements, no conclusions can be drawn on whether hermetic storage has a greater or lesser effect on seed vigor than non-hermetic storage. However, an assessment of maize at 12.5% showed that shoot and root lengths were maintained after two months of hermetic storage; while they decreased in non-hermetic storage due to an increase in m.c. [33].

### 4.2. Effect of Short-Term Hermetic Storage of Wet Grain on Other Maize Quality Attributes

There was minimal fungal growth on maize stored in the hermetic jars regardless of m.c. However, significant fungal growth was observed on maize at 21 and 24% m.c. in non-hermetic storage. The grain mass in the non-hermetic jars in this study turned black and there was a visible dust-like cloud when maize was poured out of the containers. We presume this cloud consisted of fungal spores. This is an additional indicator that there was significant mold growth in the non-hermetic jars. The results reported here are consistent with those of Williams et al. (2014) [16], who observed only very slight visible mold growth on maize stored under hermetic conditions but significant mold development on maize kept under non-hermetic conditions. In the study by Weinberg et al. (2008) [15], no visible mold was observed on maize at 18% to 22% m.c. stored under hermetic conditions after 15 days. The rapid depletion in O_2_ and increase in CO_2_ may explain the minimal development of fungi on high m.c. maize stored in hermetic jars. 

Mold growth in general and more specifically mycotoxin production, should not be an issue during hermetic storage, but a major challenge during non-hermetic storage [16]. *Aspergillus flavus*, a fungus that can grow on maize, produces aflatoxin, a carcinogenic mycotoxin. The growth of *A. flavus* after harvest can be prevented or minimized by timely drying and proper storage [6]. Instead of mold growth, it is likely that maize at 21 and 24% m.c. stored in hermetic jars underwent fermentation that caused the reduction in pH. In addition, the continual rise in CO_2_ levels in the maize at both 21 and 24% m.c. in hermetic storage, even after the O_2_ was consumed, suggests anaerobic respiration (fermentation or the activity of lactic acid bacteria). The fermentation caused by anaerobic yeast and bacteria produces primarily ethanol along with lactic acid which reduce the pH [34,35,36]. There was no obvious drop in the pH of maize samples from the non-hermetic jars.

### 4.3. Implications of Short-Term Hermetic Storage of Wet Maize on Smallholder Farms

Although there are undesirable effects of temporarily storing high-moisture maize in both hermetic and non-hermetic storage, utilizing hermetic containers provides some benefits. While changes in germination were similar at each storage time for both storage methods, hermetic storage of high m.c. maize prevented mold growth, which could eventually lead to the development of mycotoxins. A disadvantage of hermetic storage at 21% m.c. or above, in addition to germination loss, is the accumulation of byproducts of fermentation if maize is kept for a week or more. For this reason, maize should be dried as soon as possible. If drying to a safe low m.c. is not possible, partial drying to below 21% and resumption of temporary storage in hermetic conditions should maintain maize quality with minimal loss. If there is no choice but to store maize temporarily at elevated moistures before drying, hermetic would be better than non-hermetic storage because it does not lead to the accumulation of mold.

Short-term hermetic storage of high-moisture maize on smallholder farms is feasible but would require further investigation. Smallholder farmers in many developing countries have access to some forms of hermetic storage bags or airtight containers that are used to preserve grain for several months. In most cases, temporarily storing high-moisture maize in hermetic containers would not require additional investments. In this situation, the hermetic container would serve a dual purpose- preserving grain quality during the drying process (short-term storage) as well as during storage for several months (long-term storage). The short-term storage would focus more on preventing mold while maintaining germination. The long-term storage, after drying to a safe m.c., would provide the benefit of mitigating insect damage. The dual-purpose use of hermetic containers should make their purchase more attractive to smallholder farmers.

## 5. Conclusions

This study investigated short-term hermetic storage of wet maize for up to 21 days. Hermetic jars performed similarly to non-hermetic containers in preserving maize germination for up to 14 days. Germination remained above 94% for at least 14 days at 18% m.c., at least 7 days at 21% m.c., and less than 7 days at 24% m.c. for both hermetic and non-hermetic storage. Low germination rates were observed at the highest moisture content. Maize at 24% m.c. stored in hermetic jars exhibited fermentation while significant mold growth was observed in maize at 21% and 24% m.c. stored in non-hermetic jars. Maize at 18 and 21% m.c. with initial germination of >95% can be stored in hermetic containers at 25 °C for up to 14 days without a significant loss in seed viability (>90%) and minimal fermentation occurring. Additional studies are needed to assess maize quality for (i) short-term storage of wet maize in hermetic bags that are commonly used by smallholder farmers to preserve their grains, and (ii) short-term storage and drying of wet maize followed by long-term storage in hermetic technologies. The results of these studies would provide additional information required to refine the use of short-term hermetic storage of wet maize for smallholder farmers and other grain handlers.

## Figures and Tables

**Table 1:** Germination (%), shoot length (mm), and root length (mm) of maize at 18, 21, and 24% m.c. stored for up to 21 days in hermetic and non-hermetic jars at 25 °C and 80% relative humidity.

		Storage Time (Days)			
Variable	Treatment *	0 (Initial)	7	14	21
	H-m.c.18%	99.5aA **	97.07aA	95.16aA	71aB
	H-m.c.21%	97.24aA	96.44aA	92.32abA	72aB
Germination	H-m.c.24%	95.5aA	88.73bB	84.49bB	0bC
(%)	NH-m.c.18%	99.5aA	98.66aA	96.32aA	88aB
	NH-m.c.21%	97.24aA	94.32abA	94.31aA	68aB
	NH-m.c.24%	95.5aA	93abAB	83bB	4bC
	H-m.c.18%	9.44aB	21.79aA	11.90aB	16.01aAB
	H-m.c.21%	15.30aB	20.92aA	15.07aB	10.92aC
Shoot Length	H-m.c.24%	14.61aA	17.175aA	12.01aA	0bB
(mm)	NH-m.c.18%	10.32aB	18.80aA	12.64aB	13.2aAB
	NH-m.c.21%	16.48aA	35.54aA	14.75aA	11.23aA
	NH-m.c.24%	15.11aA	18.13aA	12.86aA	0bB
	H-m.c.18%	55.55aB	74.45aA	47.54aB	43.60aB
	H-m.c.21%	46.51aAB	60.89abA	42.10abA	29.51bB
Root Length	H-m.c.24%	46.17aA	48.43abA	28.42bcA	0cB
(mm)	NH-m.c.18%	55.68aA	56.96abA	40.21abA	35.0abA
	NH-m.c.21%	47.51aAA	53.74abA	41.77abAB	27.77bB
	NH-m.c.24%	57.92aA	39.57bAB	20.91cB	2.81cB

* H—Hermetic jar, NH—non-hermetic jar, and m.c.—moisture content. ** Means in the same column (lower case) and the same row (upper case) under the same variable followed by the same letters are not significantly different at *p* = 0.05.

**Table 2:** Moisture content (%) and pH of maize at 18, 21, and 24% stored for up to 21 days in hermetic and non-hermetic jars at 25 °C and 80% relative humidity.

		Storage Time (Days)			
Variable	Treatment *	0 (Initial)	7	14	21
	H-m.c.18%	17.59cA **	17.46cA	17.44cA	17.33cA
	H-m.c.21%	21.03bA	21.10bA	21.03bA	20.68bA
Moisture	H-m.c.24%	24.07aA	23.88aA	24.05aA	23.93aA
(%)	NH-m.c.18%	17.59cA	17.13cA	17.08cA	17.40cA
	NH-m.c.21%	21.03bA	20.81bA	20.84bA	20.78bA
	NH-m.c.24%	24.07aA	23.46aA	23.80aA	23.87aA
	H-m.c.18%	5.12aA	5.13bA	4.93cC	5.02cB
	H-m.c.21%	5.20aA	5.03bB	4.79dC	4.13dD
pH	H-m.c.24%	5.2aA	5.03bB	4.76dC	3.97eD
	NH-m.c.18%	5.15aA	5.27aA	5.15bA	5.15bA
	NH-m.c.21%	5.24aA	5.03bB	5.34aA	5.35aA
	NH-m.c.24%	5.25aA	5.09bB	5.15bAB	5.05bcB

* H—Hermetic jar, NH—non-hermetic jar, and m.c.—moisture content. ** Means in the same column (lower case) and the same row (upper case) under the same variable followed by the same letters are not significantly different at *p* = 0.05.

## Data Availability

The data presented in this study are available on request from the corresponding author.
